# Active breathing coordinator combines with custom-designed puncture needle improves diagnostic accuracy of ≤20 mm pulmonary nodules under CT-guidance: a real world prospective study

**DOI:** 10.3389/fonc.2025.1675444

**Published:** 2025-10-09

**Authors:** Yuting Huang, Jie Yang, Peisen Liu, Jinnan Xuan, Xin Xiao, Chao Wang, Fanliang Meng, Hao Mou, Xu Zhang, Shuang Ji

**Affiliations:** ^1^ Department of Radiotherapy, Fourth Affiliated Hospital of Anhui Medical University, Hefei, China; ^2^ Department of Respiratory and Critical Care Medicine, Fourth Affiliated Hospital of Anhui Medical University, Hefei, China; ^3^ Department of Respiratory and Critical Care Medicine, First Affiliated Hospital of Anhui Medical University, Hefei, China; ^4^ Hubei Key Laboratory of Photoelectric Materials and Devices, School of Materials Science and Engineering, Hubei Normal University, Huangshi, China; ^5^ Department of Health Management Center, First Affiliated Hospital of Anhui Medical University, Hefei, China

**Keywords:** pulmonary nodules, CT-guided percutaneous transthoracic needle biopsy, puncture needle guide, active breathing coordinator, diagnostic accuracy

## Abstract

**Background:**

CT-guided percutaneous transthoracic needle biopsy (PTNB) is still the main way for obtaining pathological diagnoses of pulmonary nodules. However, the small size and respiratory-induced motion reduce diagnostic accuracy for patients with small pulmonary nodules (≤20mm). In this study, we aimed to improve biopsy precision and diagnostic accuracy for patients with small pulmonary nodules via introducing significant refinements.

**Methods:**

122 patients with ≤20mm pulmonary nodules were enrolled and randomly assigned to the ABC-NG PTNB group and CT-guided PTNB group. The CT-guided PTNB group underwent conventional CT-guided PTNB, while the ABC-NG PTNB group received additional ways including thermoplastic immobilization, active breathing coordinator (ABC), and a custom-designed puncture needle. Puncture accuracy, diagnostic accuracy, and complication rates were compared between the two groups.

**Results:**

The ABC-NG PTNB group was superior to the CT-guided PTNB group in the terms of angle error, craniocaudal plane error, positioning error, diagnostic accuracy, and one-puncture success rate (P < 0.05). The ABC-NG PTNB group had fewer punctures, fewer CT scans, lower radiation doses, and lower incidence of pneumothorax as compared to CT-guided PTNB group (P < 0.05). Furthermore, diagnostic accuracy was particularly enhanced in cases where the puncture angle was non-zero or when the nodules were located in the lower lung lobes (P < 0.05).

**Conclusion:**

Ct-guided PTNB combined with ABC and custom-designed puncture needle guide improves the accuracy and diagnosis rate of ≤20mm pulmonary nodule biopsy, especially nodules are located in the lower lung lobe or require a non-zero puncture angle.

## Graphical Abstract

ABC-NG PTNB was superior to CT-guided PTNB for patients with ≤ 20mm pulmonary nodules.

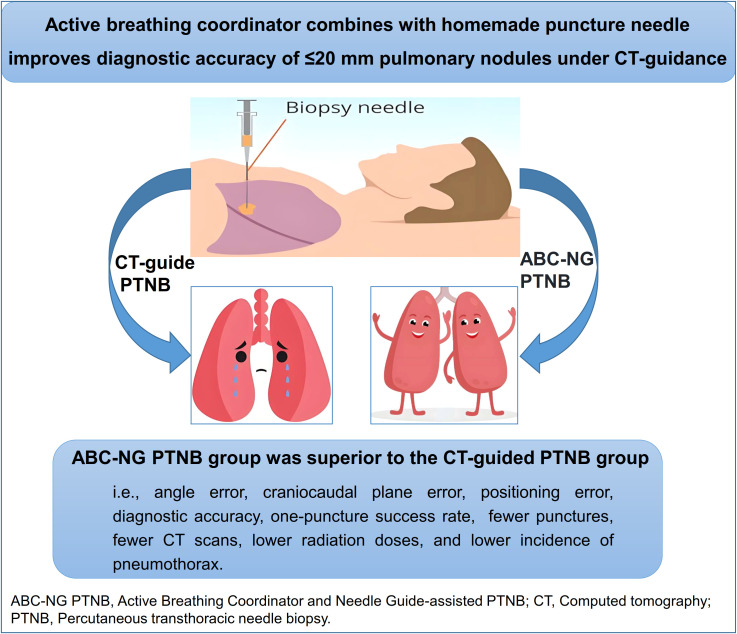

## 1 Introduction

Lung cancer persists as one of the most common malignant tumors worldwide (1), underscoring the critical importance of early diagnosis for enhancing patient outcomes. The widespread adoption of low-dose chest computed tomography (CT) in screening programs has markedly increased the detection of pulmonary nodules (2). CT-guided percutaneous transthoracic needle biopsy (PTNB) remains the primary method for obtaining pathological diagnoses of nodules (3). However, the procedure poses significant technical challenges, especially in biopsies targeting pulmonary nodules ≤20mm, due to their small size and respiratory-induced motion (4). Conventional PTNB exhibits limitations such as high operator subjectivity and inadequate control over breathing motion (5). Although various adjunctive positioning devices have been developed to address these issues (6–8), each carries inherent drawbacks. To overcome these challenges, we developed an enhanced PTNB technique termed Active Breathing Coordinator and Needle Guide-assisted PTNB (ABC-NG PTNB).

The ABC-NG PTNB integrates three key innovations. Firstly, thermoplastic immobilization. Minimizes patient shifts and involuntary movements during the procedure, ensuring stable targeting. Secondly, active breathing coordinator (ABC). Enhances biopsy accuracy by actively restricting the respiratory motion range of lung nodules. Finally, custom-designed puncture needle guide. A custom-designed needle guide (Chinese Patent ZL202221549559.6), engineered to improve upon existing technology for superior accuracy and broader applicability.

In this study, we investigated the efficacy of ABC-NG PTNB in improving biopsy precision and diagnostic accuracy for patients with small pulmonary nodules.

## 2 Subjects and methods

### 2.1 Subjects

This study received approval from the Ethics Committee of Fourth Affiliated Hospital of Anhui Medical University (Approval No.: KYXM-202201-009). From January 2022 to January 2024, 122 consecutive patients with ≤20 mm pulmonary nodules undergoing biopsy at Chaohu Hospital of Anhui Medical University were enrolled. Participants were randomized into two groups: the ABC-NG PTNB group (n=61) and the conventional CT-guided PTNB group (n=61).

### 2.2 Inclusion criteria

Patients were enrolled according to the following criteria: (1) Presence of pulmonary nodules (≤20 mm) classified as Lung-RADS category 4 on CT imaging (9). (2) Full capacity to cooperate during the procedure. (3) Absence of major blood vessels or bullae along the biopsy trajectory. (4) No significant organ dysfunction. (5) Normal baseline findings in complete blood count, electrocardiogram, and coagulation tests.

### 2.3 Procedural methods

#### 2.3.1 Key approaches in ABC-NG PTNBT hermoplastic immobilization

**Figure 1:**
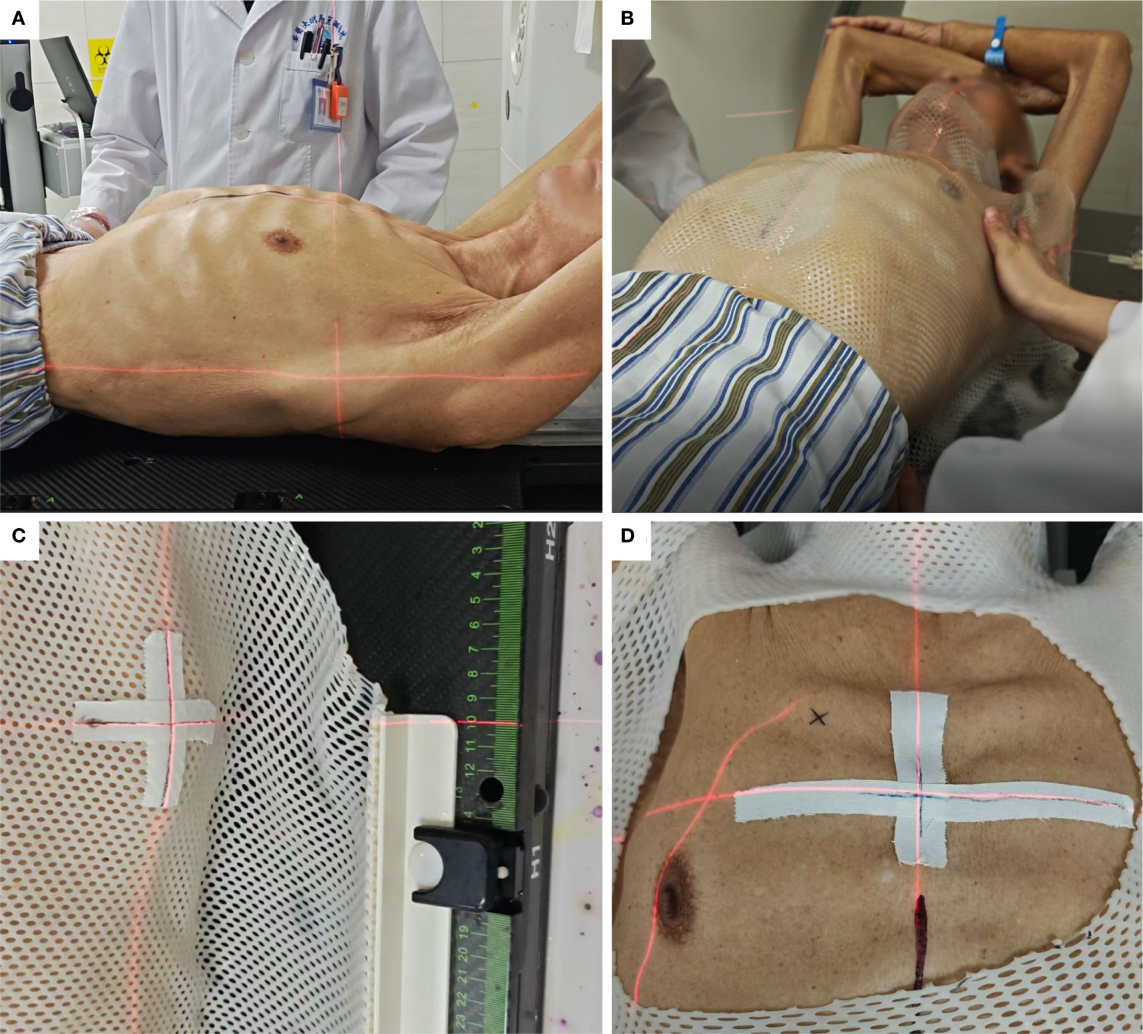
Thermoplastic immobilization. **(A)** Selection of patient position. **(B)** Moulding of the thermoplastic membrane. **(C)** Cross-marking to monitor positional accuracy. **(D)** Window creation around the puncture site.

Patients were optimally positioned by two operators. A thermoplastic membrane was heated to 66–72 °C until pliable, molded onto the skin surface, and allowed to solidify for positional stability. An access window was subsequently created at the lesion site (**Figure 1**).

(1) Active Breathing Coordinator (ABC)

**Figure 2:**
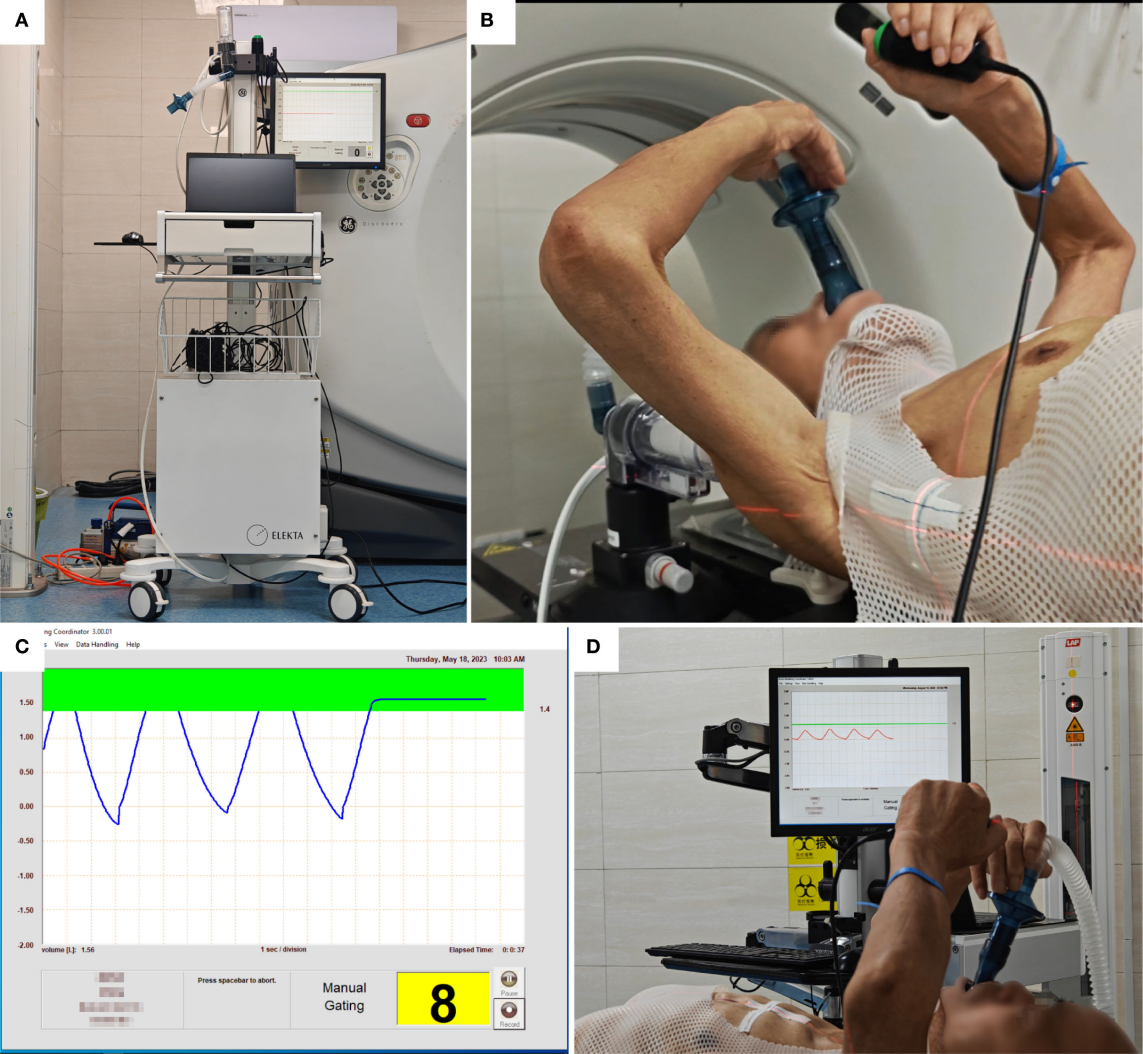
Active breathing coordinator (ABC) training. **(A)** ABC system. **(B)** Breathing training, press the button system runs, release the button system stops. **(C)** Breathing cycle, press the control button when holding breath, is pressed during breath-hold, indicated by a green bar on the monitor; the system closes the valve when the inhaled air volume reaches the upper limit of the green bar. **(D)** ABC breath-hold training sequence.

The Elekta ABC system (Sweden) facilitated deep inspiration breath-hold (DIBH). Patients breathed freely until instructed to inspire a predetermined tidal volume, after which the system automatically occluded airflow. This standardized lung volume ensured consistent nodule positioning during biopsy (**Figure 2**).

(2) Custom-designed puncture needle guide

**Figure 3:**
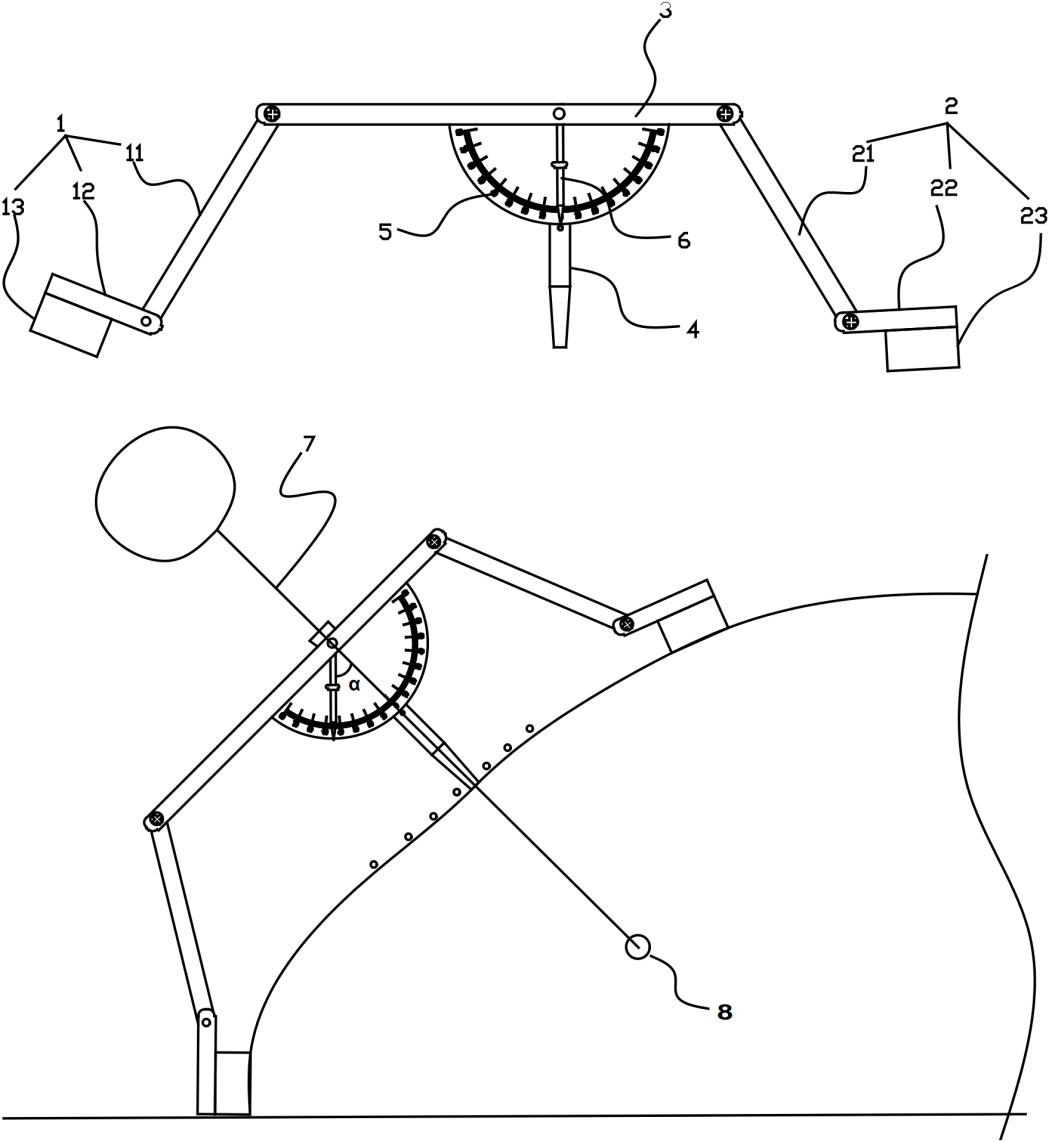
Components of the custom-designed CT puncture needle guide. (1) First leg assembly. (11) First leg, (12) First fixing plate, (13) First adhesive layer. (2) Second leg assembly. (21) Second leg, (22) Second fixing plate, (23) Second adhesive layer. (3) Positioning plate. (4) Positioning tube. (5) Scale plate. (6) Pointer. (7) Puncture needle. (8) Pulmonary nodule.

A custom-designed needle guide (**Figure 3**, Chinese Patent ZL202221549559.6) was developed to address limitations of commercial devices. Key features include: (1) Integrated gravity indicator maintaining perpendicularity to the horizontal plane. (2) Correction of angular deviation, trajectory misalignment, needle instability, and depth inaccuracy. (3) Streamlined design offering enhanced usability, procedural efficiency, and cost-effectiveness.

#### 2.3.2 Procedural workflow

**Figure 4:**
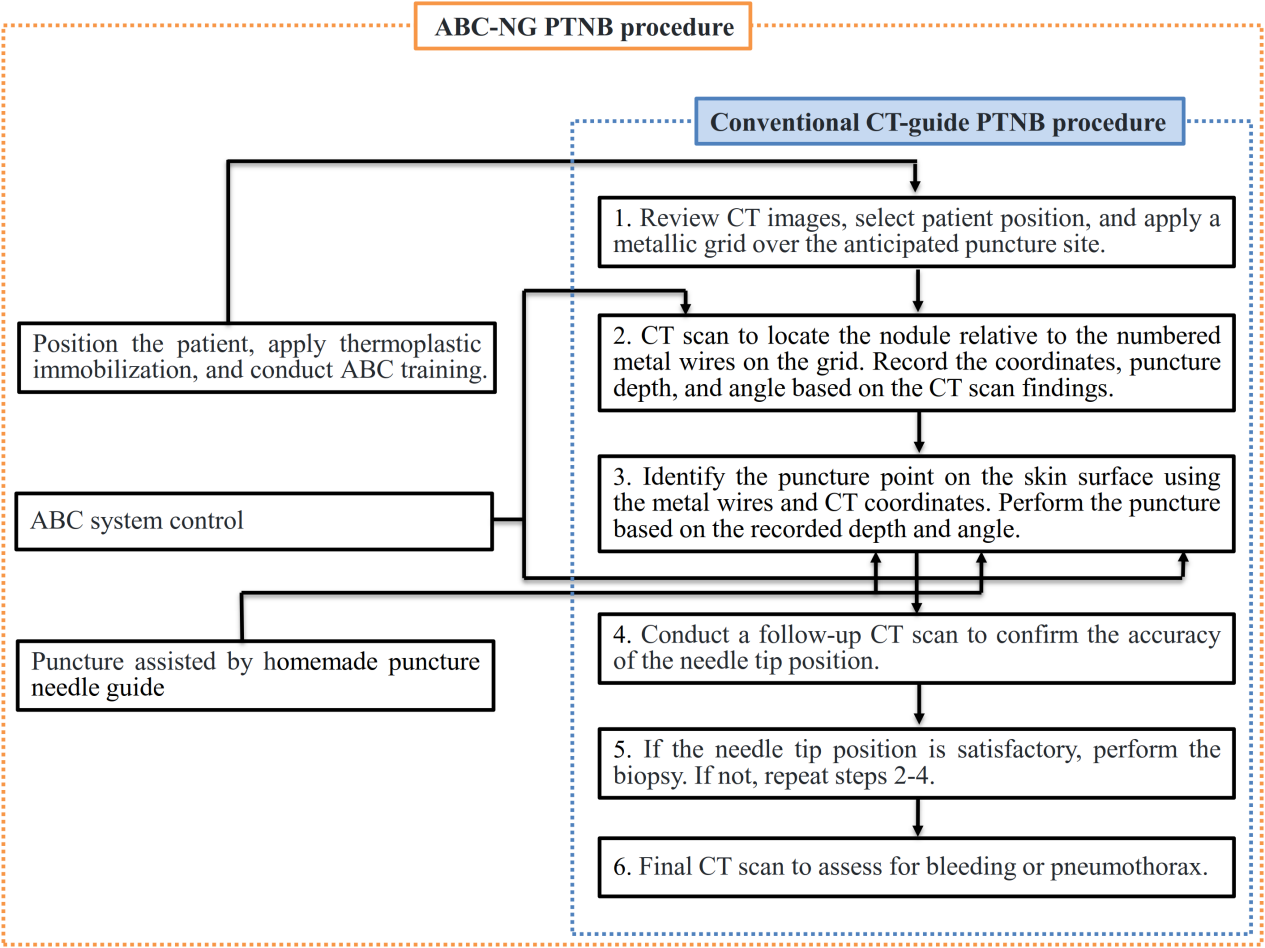
Procedure flow and differences between the two groups.

Both traditional CT-guided PTNB group and ABC-NG PTNB group procedures were performed by two associate-chief physicians (>10 years of biopsy experience). Key procedural distinctions and similarities are illustrated in **Figure 4**.

The traditional CT-guided PTNB group followed the Chinese multidisciplinary expert consensus: Guidelines for percutaneous transthoracic needle biopsy (10). The procedure utilized a large-bore CT scanner (Discovery RT, GE Healthcare, USA) and a laser positioning system (DORADOnova 5, LAP, Germany). Pre-biopsy CT localized the tumor region. A custom-made metal grid was affixed to the skin surface above the tumor site, and a CT scan was performed to determine the relative position of the tumor to the corresponding numbered metal wire on the grid. The puncture plane, distance, and angle between the metal wire and the tumor were measured. The puncture site was determined on the skin surface using the metal wire and CT coordinates, and the laser positioning system was used to move the bed and mark the puncture site. After applying 5% lidocaine for local anesthesia, a 17G coaxial puncture needle (TUORen, China) was used to perform the biopsy according to the measured distance and angle. Another CT scan was performed to verify the needle’s position. If the needle was not in the intended location, adjustments were made; if it was correctly positioned, an 18G semi-automatic biopsy needle (TSK, Japan) was used to obtain tissue samples. The biopsy tissue was examined visually; if satisfactory, 1–2 additional samples were taken; otherwise, the needle position was adjusted for a repeat biopsy. A post-procedure CT scan was conducted to assess for complications such as bleeding or pneumothorax.

In the ABC-NG PTNB group, patients underwent DIBH training prior to the procedure to ensure they could cooperate with controlled breathing. After training, the appropriate position was selected for the patient, and thermoplastic immobilization (thermoplastic film model: RF-V106-2001W) was applied. The CT scan was reviewed to identify the location on the thermoplastic membrane where a window was created for the skin puncture site. Before the CT scan, the ABC system was activated to control DIBH, with the scan initiated immediately after breath-hold. The puncture site was selected based on the scanned CT images, and the ABC system was reactivated prior to the biopsy to ensure the nodule returned to its position within the lungs during inhalation. The custom-designed CT puncture needle guide was used to precisely control the puncture distance and angle.

**Figure 5:**
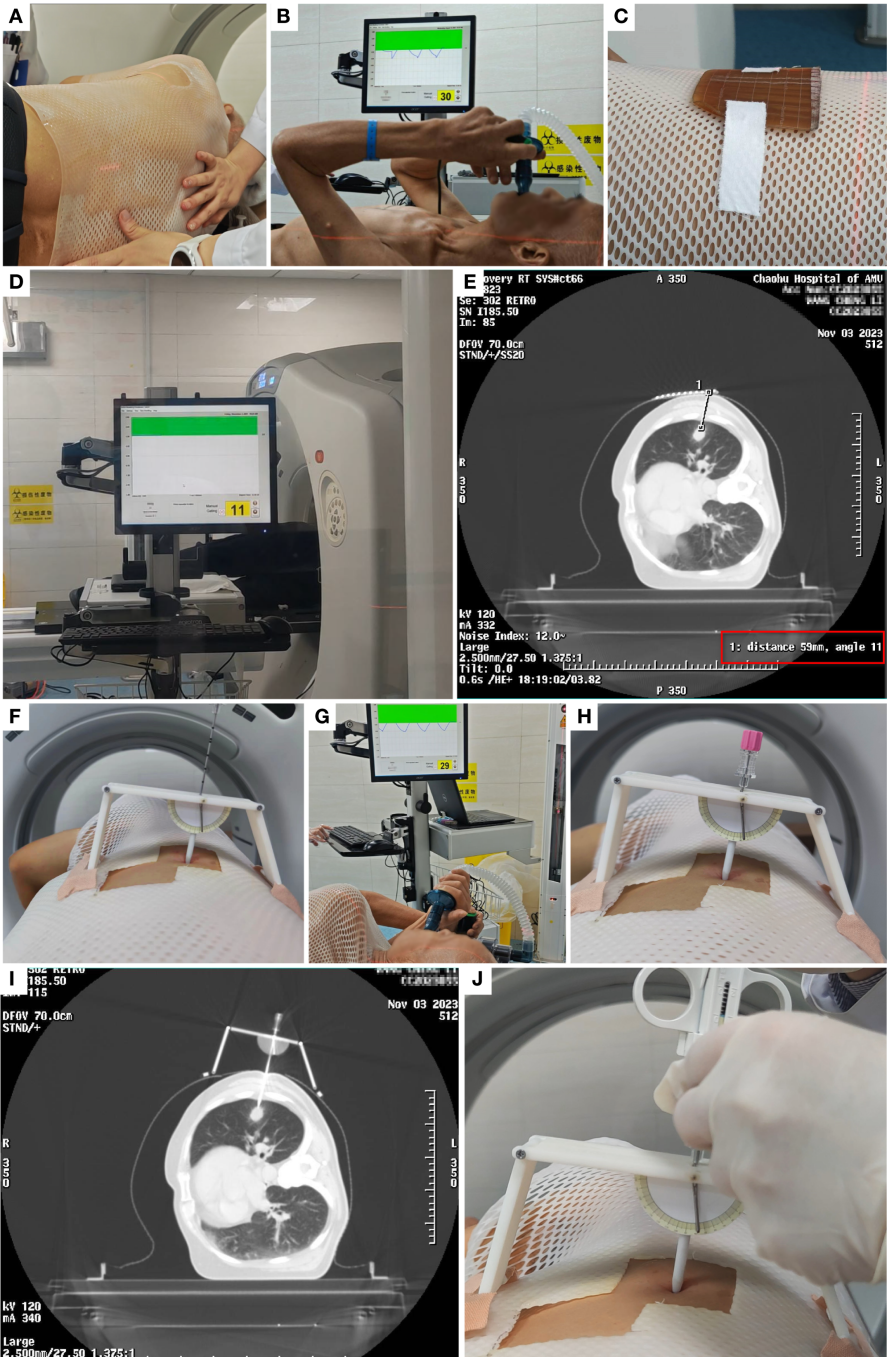
Example of the procedure flow in observation group. **(A)** Thermoplastic Immobilization. **(B)** ABC training. **(C)** Metallic grid. **(D)** ABC-guided CT scanning. **(E)** Determine the CT slice, angle, and depth for puncture. **(F)** Identify the puncture point using coordinates and grid, set the angle on the needle guide, and secure it. **(G-H)** Perform puncture under ABC guidance. **(I)** Re-scan with CT to confirm puncture success. **(J)** Conduct biopsy after successful puncture.

The differences and similarities between the two procedures are illustrated in **Figure 4**, and the specific workflow for the ABC-NG PTNB group is detailed in **Figure 5**.

### 2.4 Data collection

#### 2.4.1 General patient data and technical parameters

(1) Baseline Characteristics: Patient sex, age, pulmonary nodule size (mm), and anatomical location. (2) Angular Deviation: Absolute difference (°) between planned and achieved needle trajectories on CT imaging. (3) Depth Discrepancy: Absolute difference (mm) between planned target depth and final needle tip position. (4) Cranio-caudal Plane Offset: Deviation from target puncture plane quantified in number of CT slices (2.5 mm slice thickness), where positive values indicate caudal displacement. (5) 3D Positional Variance: Euclidean distance (mm) between preoperative and intraoperative skin fiducial markers in three-dimensional coordinates (X, Y, Z axes).

#### 2.4.2 Clinical outcomes

(1) Diagnostic Accuracy: Proportion of biopsies yielding definitive pathological diagnosis (benign/malignant) confirmed by reference standard (surgical histopathology, ≥6-month imaging follow-up, or alternative diagnostic verification), excluding non-diagnostic samples and normal parenchyma. (2) First-Pass Success Rate: Proportion of procedures where the needle tip achieved intranodular positioning on initial insertion, confirmed by immediate CT verification. (3) Puncture Attempt Frequency: Total count of distinct needle insertions or major trajectory adjustments (>5° angular or >5 mm depth modification) required to obtain adequate samples. (4) Total Procedure Time: Duration (minutes) from initial patient positioning to removal of all biopsy equipment, including preparatory steps. (5) Core Biopsy Time: Duration (minutes) from first localization CT scan to final tissue acquisition completion. (6) Cumulative CT Dose: Total radiation exposure quantified as Dose-Length Product (DLP, mGy·cm) across all intraprocedural scans. (7) Complication Incidence: Proportion of procedures resulting in procedure-related adverse events (e.g., pneumothorax requiring intervention, hemoptysis >50 mL), graded per CIRSE classification.

### 2.5 Statistical analysis

Data analysis was conducted using SPSS 26.0 software. Continuous data with normal distribution were expressed as mean ± standard deviation and compared using independent sample t-tests. Non-normally distributed variables were expressed as Median (interquartile range [IQR]) and analyzed using Mann-Whitney U test. Categorical data were compared using chi-square test (χ²) or fisher’s exact test. Significance level was set at α = 0.05.

## 3 Results

### 3.1 Patient cohort

A total of 122 patients were randomized to the ABC-NG PTNB group (n=61) and the CT-guided PTNB group (n=61). In the ABC-NG PTNB group, there were 44 males and 17 females, with a median age of 67 years (range: 26-76) and median nodule size 18 mm (range: 11-20). In the CT-guided PTNB group, 42 males and 19 females, with a median age of 68 years (range: 21-76) and median nodule size 18 mm (range: 14-20). Baseline characteristics were comparable between groups (all *P*-values > 0.05, **Table 1**).

**Table 1:** Comparison of general characteristics between the two groups.

Characteristic	ABC-NG PTNB group (n=61)	CT-guided PTNB group (n=61)	*Z/t/χ* ^2^	*P-value*
Age (years)	67(56;72.5)	68(58.5;73)	-0.528	0.597
Gender			0.158	0.691
- Male	44(72.1%)	42(68.9%)		
- Female	17(27.9%)	19(31.1%)		
Patient position			0.229	1.000**
- Supine	26(42.6%)	25(41.0%)		
- Prone	32(52.5%)	32(52.5%)		
- Decubitus	3(4.9%)	4(6.5%)		
Nodule size (mm)	18(17;19)	18(18;19)	-0.342	0.733*
Nodule depth from chest wall (mm)	28.6 ± 13.7	30.3 ± 14.1	-0.683	0.496
Nodule location			0.033	0.856
- Upper and middle lobes	28(45.9%)	29(47.5%)		
- Lower lobes	33(54.1%)	32(52.5%)		

*: Mann-Whitney U test **: fisher’s exact test

### 3.2 Technical performance

The ABC-NG PTNB group showed significantly reduced angular deviation, cranio-caudal plane offset, and 3D positional variance compared to the CT-guided PTNB group (all *P*-values < 0.05, **Table 2**). No significant difference in angular error at 0° puncture angle (Z = -1.602, *P* = 0.109, **Table 2**). Whereas, significantly lower angular error at non-zero angles (Z = -6.568, *P* < 0.001, **Table 2**). Furthermore, ABC-NG PTNB required fewer puncture attempts, reduced CT scans, and lower cumulative radiation doses (DLP) (all *P*-values < 0.001, **Table 2**).

**Table 2:** Comparison of technical parameters between the two groups.

Parameters	ABC-NG PTNB group (n=61)	CT-guided PTNB group (n=61)	*Z/t*	*P-value*
Angle Error (°)	3(2;4)	6(3;9)	-5.498	<0.001*
- Puncture Angle at 0°	2(2;3)	3(2;4)	-1.602	0.109*
- Puncture Angle not at 0°	3(2;4)	8(6;10)	-6.568	<0.001*
Depth Error (mm)	4(3;5)	4(3;6)	-0.761	0.446*
Craniocaudal Plane Error (Slices)	3(2;4)	4(2;5)	-2.285	0.022*
Positional Error (mm)	39.0 ± 9.6	61.6 ± 15.5	-9.672	<0.001
Number of Punctures (times)	2(1;2)	3(2;3)	-4.845	<0.001*
Number of Scans (times)	5(4;5)	6(5;6)	-4.803	<0.001*
Radiation doses (DLP, mGy·cm)	876 ± 270	1213 ± 321	-6.282	<0.001

DLP, dose length product *: Mann-Whitney U test.

### 3.3 Clinical outcomes

The diagnostic accuracy and one-puncture success rate were both significantly higher in the ABC-NG PTNB group as compared to the CT-guided PTNB group (both *P* < 0.05, **Table 3**). There was no significant difference in the total operation time between the two groups (t = 0.630, *P* = 0.530, **Table 3**). However, when the time for thermoplastic immobilization and ABC training was excluded, the puncture biopsy time in the ABC-NG PTNB group was significantly shorter than that in the CT-guided PTNB group (16.7 ± 3.2 minutes vs. 23.4 ± 6.2 minutes; t = -7.587, *P* < 0.001, **Table 3**). The incidence of pneumothorax was 16.4% in the ABC-NG PTNB group and 32.8% in the CT-guided PTNB group, with the ABC-NG PTNB group showing a significant lower rate (χ² = 4.420, *P* = 0.036, **Table 3**). The incidence of hemoptysis was lower in the ABC-NG PTNB group compared to the CT-guided PTNB group, but did not reach statistical significance (χ² = 3.055, *P* = 0.081, **Table 3**).

**Table 3:** Comparison of clinical outcomes between the two groups.

Characteristic	ABC-NG PTNB group (n=61)	CT-guided PTNB group (n=61)	*t/χ* ^2^	*P-value*
Diagnostic Accuracy	54/61(88.5%)	44/61(72.1%)	5.187	0.023
One-puncture success rate	52/61(85.2%)	35/61(57.4%)	11.579	0.001
Total Procedure Time (minutes)	24.0 ± 3.1	23.4 ± 6.2	0.630	0.530
Puncture Biopsy Time (minutes)	16.7 ± 3.2	23.4 ± 6.2	-7.587	<0.001
Complications				
- Pneumothorax	10/61(16.4%)	20/61(32.8%)	4.420	0.036
- Hemoptysis	6/61(9.8%)	13/61(21.3%)	3.055	0.081

### 3.4 Stratified analysis of diagnostic accuracy

When the puncture angle was non-zero or the lung nodule was located in the lower lobes of the lungs, the diagnostic accuracy in the ABC-NG PTNB group was significantly higher than that in the CT-guided PTNB group (**Table 4**). When the puncture angle was zero degree or the lung nodule was located in the upper or middle lobes of the lung, the diagnostic accuracy of the ABC-NG PTNB group was higher than that of the CT-guided PTNB group, but the difference was not statistically significant (**Table 4**).

**Table 4:** Stratified analysis of diagnostic accuracy between the two groups.

Characteristic	Diagnostic success	Diagnostic failure	χ^2^	*P-value*
Puncture angle at 0°				
- ABC-NG PTNB group	22(88.0%)	3(12.0%)	–	0.463*
- CT-guided PTNB group	19(76.0%)	6(24.0%)		
Puncture angle not at 0°				
- ABC-NG PTNB group	32(88.9%)	4(11.1%)	4.126	0.042
- CT-guided PTNB group	25(69.4%)	11(30.6%)		
Nodule location: upper or middle lobe				
- ABC-NG PTNB group	26(92.9%)	2(7.1%)	–	0.423*
- CT-guided PTNB group	24(82.8%)	5(17.2%)		
Nodule location: lower lobe				
- ABC-NG PTNB group	28(84.8%)	5(15.2%)	4.201	0.040
- CT-guided PTNB group	20(62.5%)	12(37.5%)		

*: fisher’s exact test.

## 4 Discussion

CT-guided PTNB remains the primary procedure of pathological diagnosis of peripheral pulmonary nodules (11). However, technical limitations persist for small nodules (≤20 mm) (12), where success heavily depends on operator skill. Performing biopsies on small pulmonary nodules presents several technical challenges. Patient movement during the procedure often causes positional inaccuracies, compromising targeting precision. While standard practice involves positioning patients comfortably to minimize motion, this approach may restrict optimal biopsy positioning. The resulting trade-off between patient comfort and procedural accuracy remains a persistent clinical dilemma. Secondly, controlling the needle insertion angle remains technically challenging. To address the issue, Jeon et al. (13) improved precision by implementing a laser guidance system for CT-guided lung biopsies, reporting 96.2% diagnostic accuracy for pulmonary lesions measuring 10–70 mm in diameter. Similarly, Xu et al. (14) similarly demonstrated that a laser-guided angle selection system enhanced both first-pass success rates and overall accuracy in percutaneous lung biopsies compared to conventional approaches. These methods nevertheless share a critical limitation, i.e., they cannot compensate for craniocaudal plane deviations, potentially causing the needle to miss its intended target by entering adjacent anatomical planes. Finally, respiratory-induced nodule movement. Small pulmonary nodules can move with respiration, especially those located in the lower lobes of the lungs. The range of movement can exceed the size of the nodule, with movements of greater than 20 mm reported (15), which is a main reason of biopsy failure. In conventional biopsy procedures, physicians usually instruct patients to inhale and hold their breath prior to the puncture. However, inconsistencies in the amount of air inhaled by the patient, as well as potentially involuntary breathing, can lead to the displacement of the nodule during the procedure. This movement further lead to the puncture needle into the pleura, increasing the risk of pneumothorax. Enrique et al. (16) addressed the problem of lung nodule movement by implementing a technique combining positive airway pressure with paused mechanical ventilation during lung cancer ablation. This approach effectively reduced nodule motion but also introduced a serious life-threatening risk. Kim et al. (17) used moderate sedation with electromagnetic navigation for PTNB, which reduce nodule motion but the diagnostic accuracy was 75%. Given the limitations of the above procedure, a safer and more effective PTNB is urgently needed.

The ABC-NG PTNB significantly improves upon the limitations of existing PTNB, offering several key advantages and innovations. Firstly, thermoplastic immobilization for enhanced stability. We used thermoplastic immobilization, a method commonly used in precise radiotherapy, to ensure a stable patient position throughout the biopsy. The restraint provided by the thermoplastic membrane assists the patient in maintaining a consistent position on the operating table for extended periods, thereby reducing positional errors during lengthy procedures (18). Additionally, the membrane restricts thoracic movement, resulting in reduced amplitude of each respiration. Consequently, the application of this method enhances the safety and precision of the procedure. Furthermore, the total duration of the puncture procedure was approximately 24 minutes (with the puncture itself taking 16 minutes and postural fixation and training taking 8 minutes), indicating that this method does not increase patient waiting time or physician workload. In this study, patients with thermoplastic immobilization had a significantly smaller total displacement error (39.0 ± 9.6 mm) compared to patients without thermoplastic immobilization (61.6 ± 15.5 mm) (t = 9.672, *P* < 0.001). Secondly, improved accuracy with a custom-designed puncture needle guide. We developed a custom-designed puncture needle guide with higher spatial accuracy in our ABC-NG PTNB group. Specifically, the puncture angle error was reduced to 3 (2, 4) in the ABC-NG PTNB group, compared to 6 (3, 9) in the CT-guided PTNB group, and the craniocaudal plane error was also reduced (3 (2, 4) vs. 4 (2, 5)). Unlike conventional laser positioning systems (13, 14), this approach aligns the vertical groove of the needle guide with the CT chamber, ensuring that the needle is perpendicular to the skin and thus reducing craniocaudal errors. Thirdly, streamlined procedures and reduce resource consumption. Current diagnostic success rate is rapid on-site evaluation (ROSE) (4), where pathologists assess specimen quality in real time. However, ROSE is labor-intensive and time-consuming. In contrast, ABC-NG PTNB streamlines the procedure, reducing puncture biopsy time from 23.4 ± 6.2 minutes in the CT-guided PTNB group to 16.7 ± 3.2 minutes in the ABC-NG PTNB group (t = -7.587, *P* < 0.001). This efficiency not only saves resources but also makes the procedure more accessible, allowing even novice practitioners to achieve proficiency with minimal practice. Future studies will further investigate the performance of beginners using this approach. Finally, addressing respiratory-induced movement using ABC. Inspired by techniques used in lung cancer radiotherapy (19), we integrated ABC into PTNB. This approach stabilizes internal thoracic positioning by converting the patient’s respiratory airflow into an electrical signal, which is then used to generate a breath-holding state at a set inspiratory threshold. In this study, the one-puncture success rate was significantly higher in the ABC-NG PTNB group (85.2%) compared to the CT-guided PTNB group (57.4%) (*P* = 0.001). Importantly, this approach avoids the risks associated with procedures like “ continuous positive airway pressure under sedation” which can endanger patients’ lives. Furthermore, the incidence of pneumothorax was significantly lower in the ABC-NG PTNB group (*P* = 0.036). Although the reduction in hemoptysis incidence did not reach statistical significance (*P* = 0.081), this might be due to the limited sample size.

Compared to previous studies (13, 14, 20), our research yielded several new findings. Firstly, when the puncture path was set at 0 degrees, the angle errors in the ABC-NG PTNB and CT-guided PTNB groups were 2 (2, 3) and 3 (2, 4), respectively, with no significant difference (*P* = 0.109). However, for the non-zero angles, the angle errors were 3 (2, 4) in the ABC-NG PTNB group vs. 8 (6, 10) in the CT-guided PTNB group, respectively, indicating that the needle guide significantly reduced positional errors (*P <*0.001). Despite the use of needle guide, angle errors were not completely eliminated, potentially due to skin resistance and needle tremors during the puncture process. Secondly, this study also found significant differences in one-puncture success rates and diagnostic accuracy between the two groups. The diagnostic accuracy for pulmonary nodules with a diameter ≤2 cm in the two groups was 88.5% and 72.1% (*P* = 0.023), respectively, which is similar to the findings of Lee et al. (21). However, when the puncture angle was 0 degree or when the pulmonary nodule was located in the upper or middle lobes of the lung, the diagnostic accuracy of the ABC-NG PTNB group, although higher than that of the CT-guided PTNB group, did not reach statistical significance. This might be due to the CT-guided PTNB group increasing the number of punctures when initial samples were unsatisfactory, thereby achieving higher diagnostic accuracy. In situations where the puncture angle was non-zero or the pulmonary nodules were located in the lower lobes, the ABC-NG PTNB resulted in higher diagnostic accuracy, fewer punctures, and lower complication rates. This suggests that the improved procedure is particularly effective in more challenging puncture situations, such as non-zero angle punctures or nodules located in the lower lobes of the lungs. ABC-NG PTNB, integrating respiratory control, effectively minimizes errors stemming from tumor motion near the diaphragm, particularly in the lower lungs and liver. This improvement significantly increases the precision of needle placement in these challenging regions, thus reducing the need for repeated punctures and associated complications.

We aim to extend the application of ABC-NG PTNB across a broader range of clinical scenarios. For instance, small-volume lung cancers and pulmonary metastases in the lower lungs, characterized by significant respiratory movement, often complicate ablation therapy (22). ABC-NG PTNB has the potential to improve the outcomes of such therapies. Additionally, preoperative localization of pulmonary nodules, a critical step prior to surgery (23), could be performed with greater accuracy and efficiency using this approach. For liver tumors near the diaphragm, which are also subject to respiratory motion, ablation is typically guided by real-time ultrasound. However, when lesions are not visible on ultrasound, CT-guided puncture is required (24). ABC-NG PTNB may improve the accuracy of these procedures as well. Similarly, during brachytherapy seed implantation for small pulmonary metastases, the small size of the lesions often hampers precise needle placement, necessitating multiple CT scans and repeated adjustments, which increase both radiation exposure and the risk of lung injury (25). By improving initial accuracy, ABC-NG PTNB could reduce these complications.

Some limitations should be considered. Some elderly patients, due to advanced age or limited education, may have difficulty following breathing instructions, limiting the applicability of ABC-NG PTNB. Furthermore, the custom-designed puncture needle guide has a fixed channel length, restricting the depth of needle insertion, which can pose challenges for accessing deeply situated tumors or patients with major blood vessels/pulmonary bullae along the intended path. Future developments will focus on creating guides with adjustable channel lengths to address this issue.

ABC-NG PTNB improved the puncture accuracy of pulmonary nodules with a diameter of ≤20 mm, especially the diagnostic accuracy of nodules located in the lower lobes or requiring non-zero-degree puncture, while reducing the incidence of puncture-related complications.

## Data Availability

The original contributions presented in the study are included in the article/supplementary material. Further inquiries can be directed to the corresponding authors.
